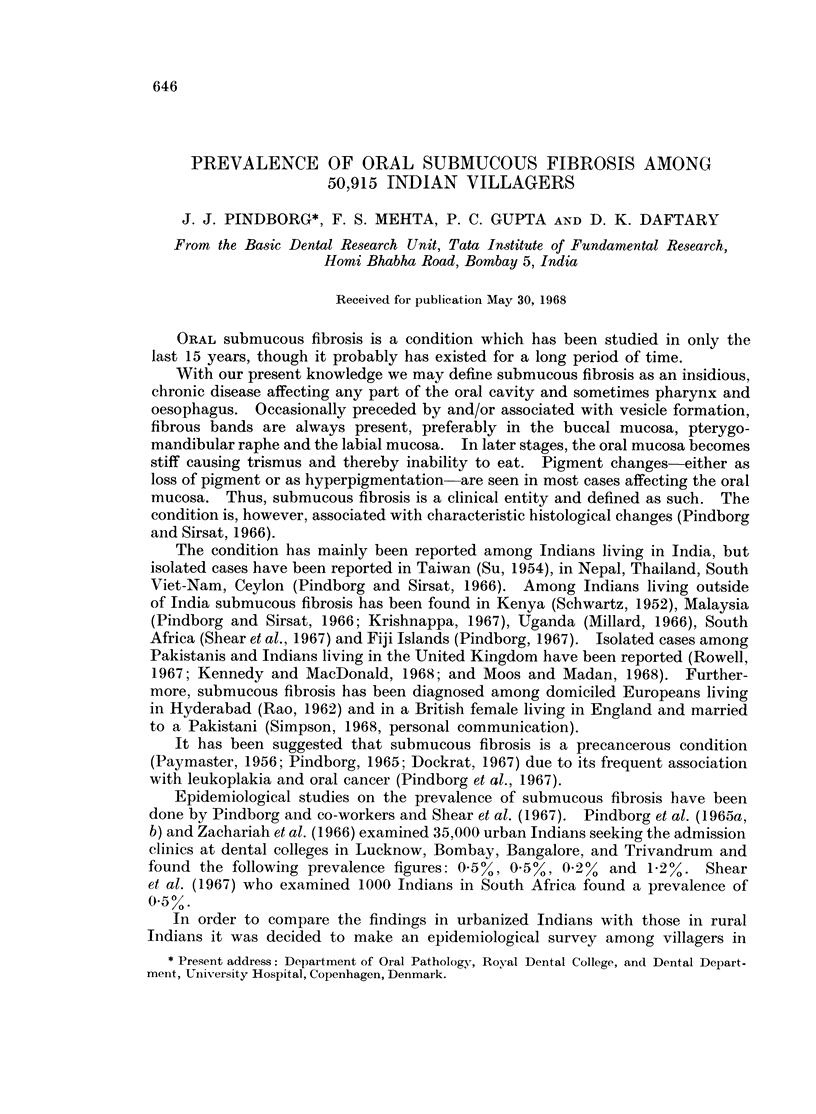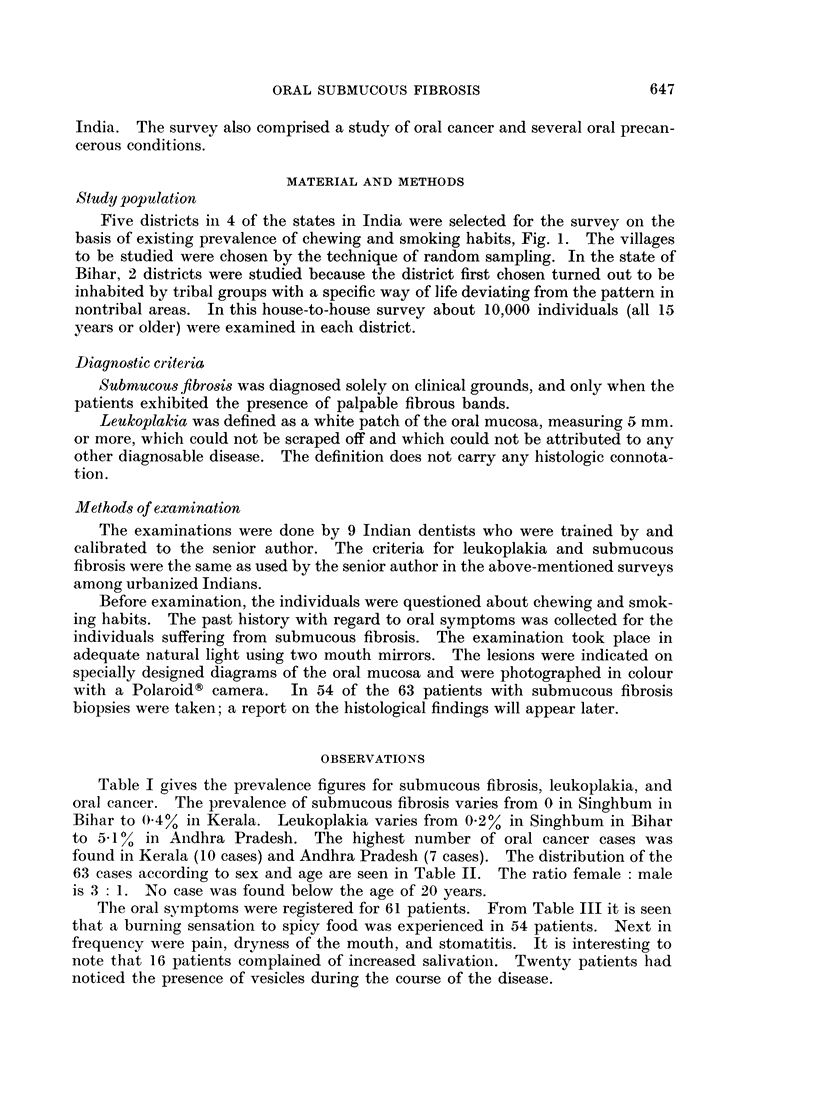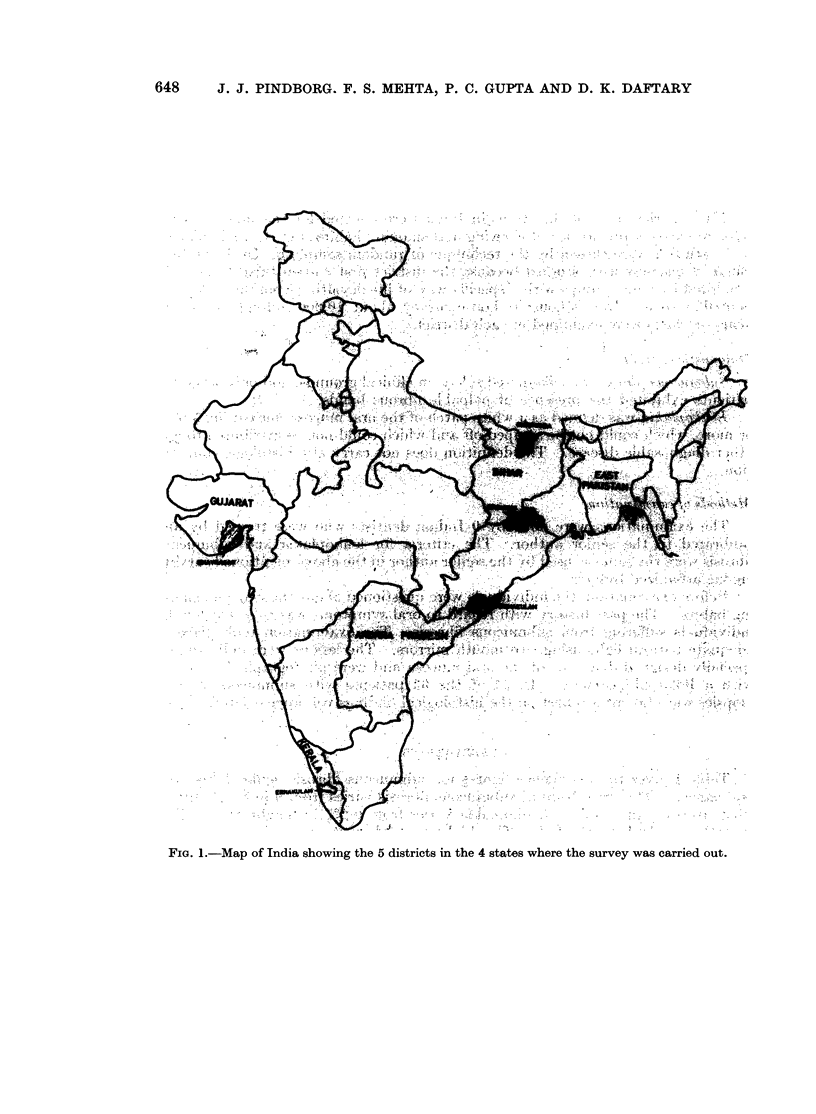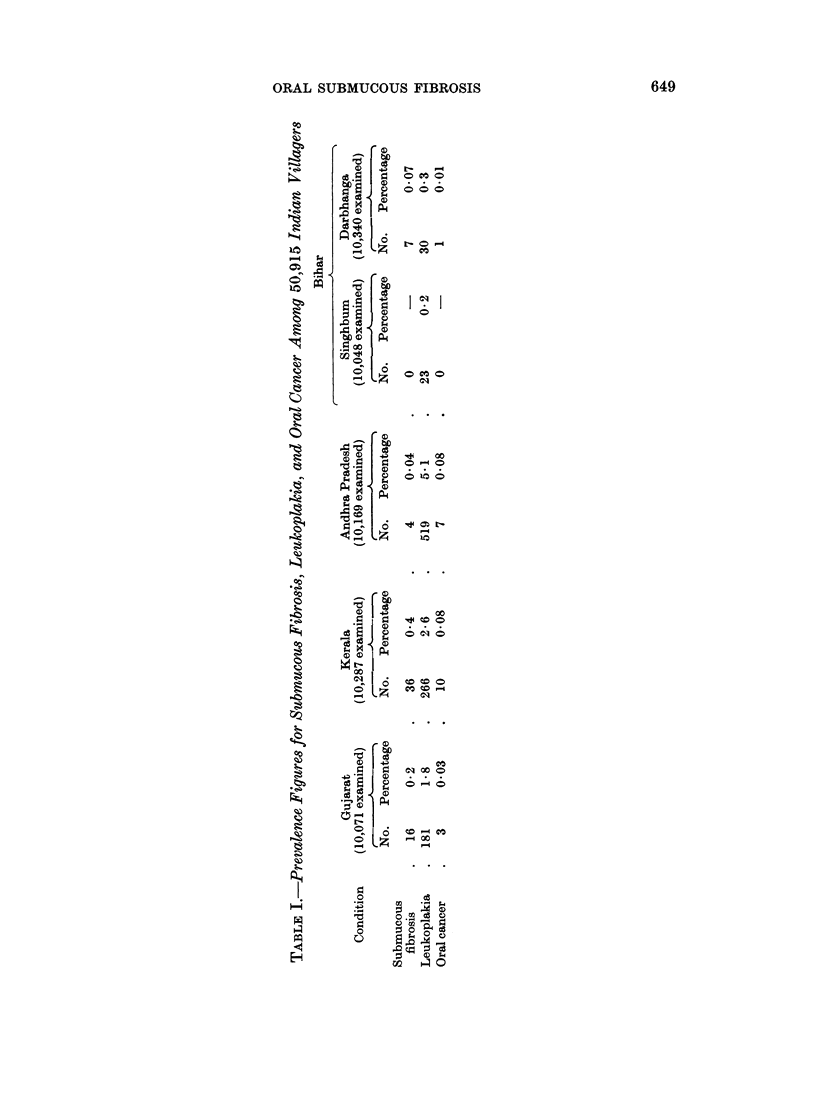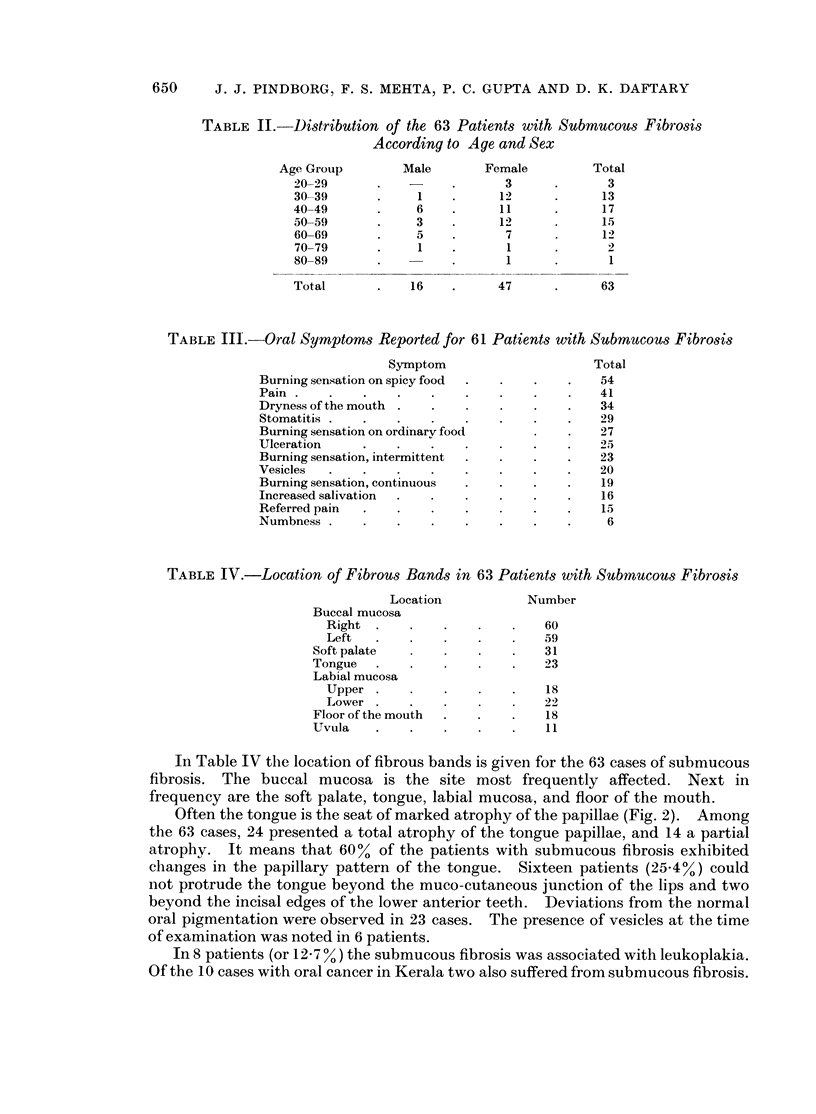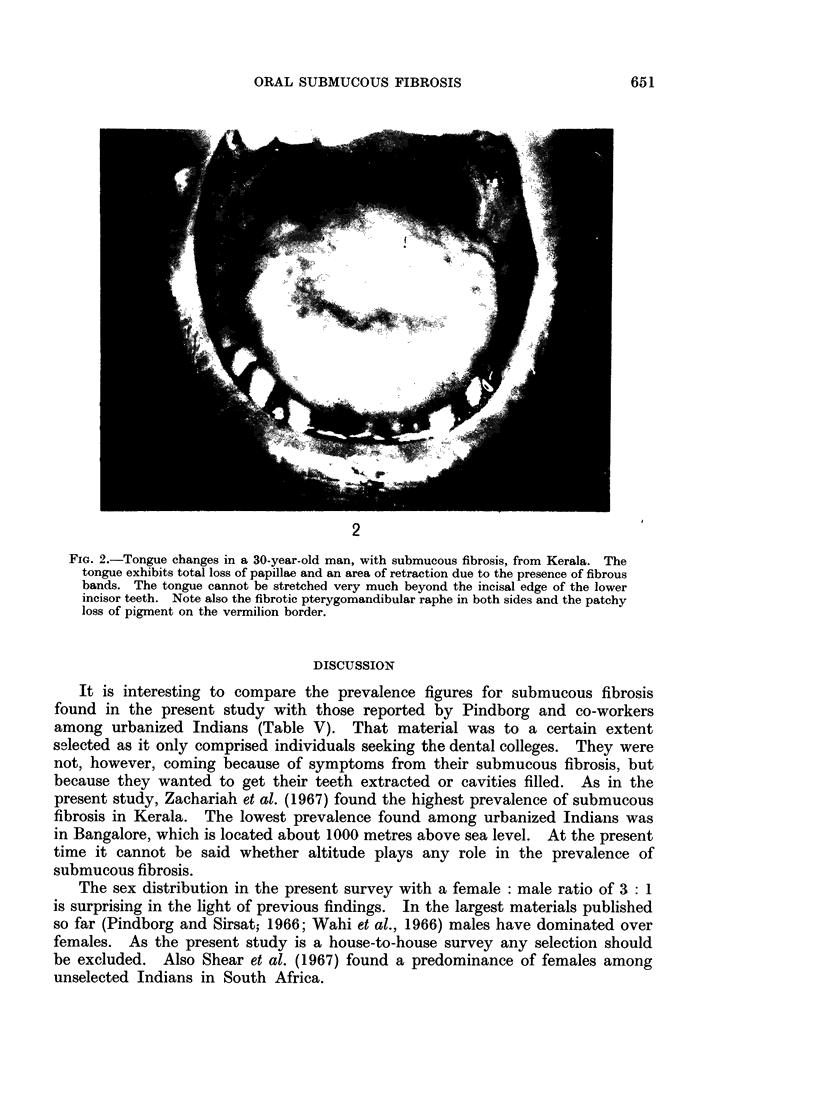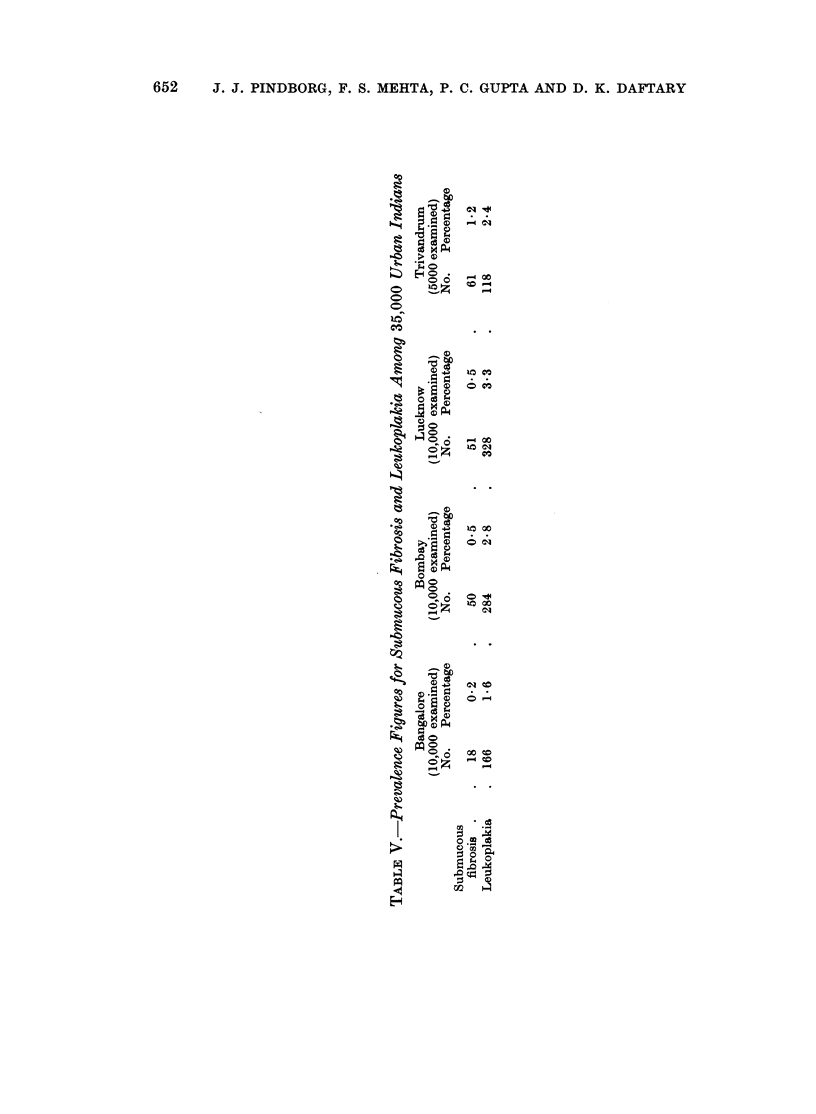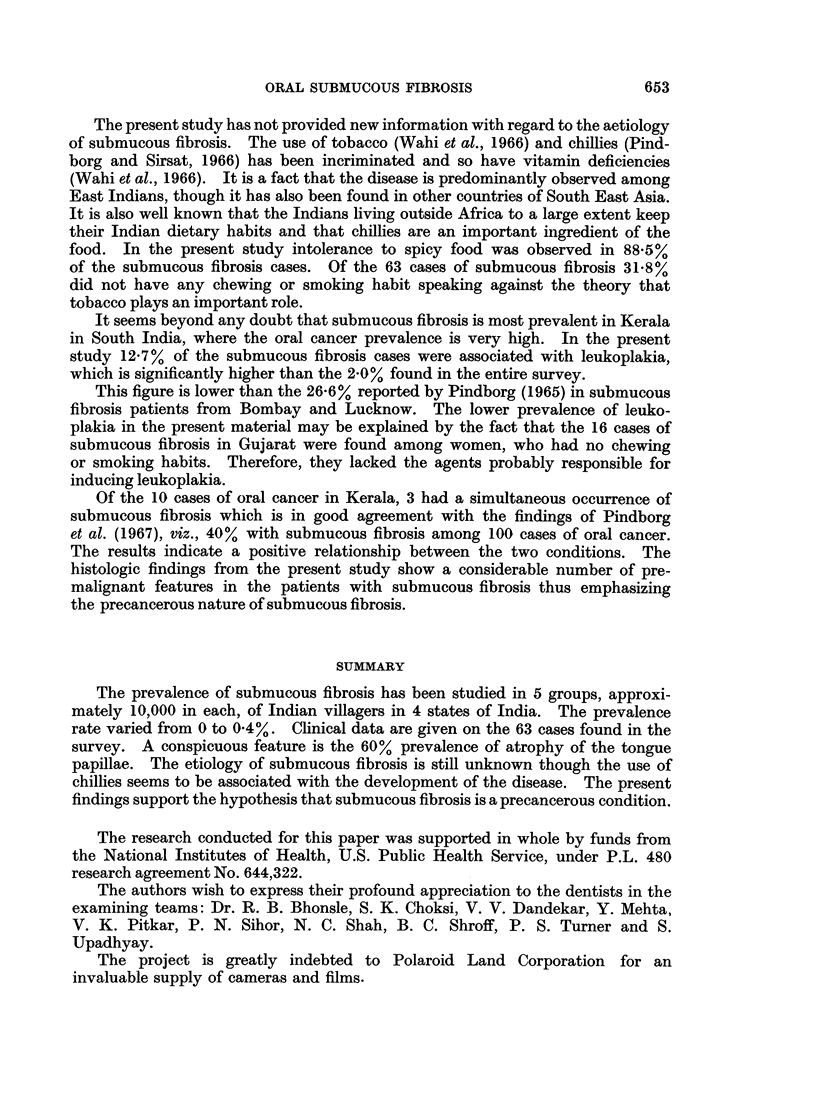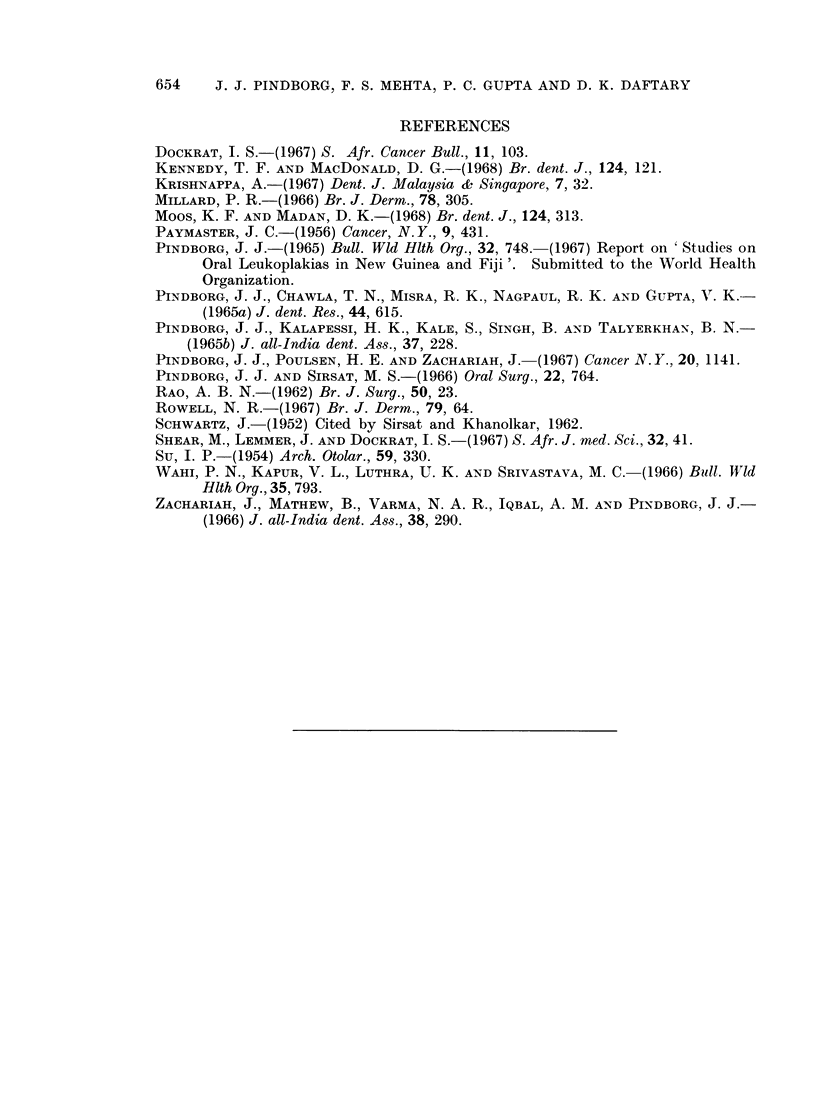# Prevalence of oral submucous fibrosis among 50,915 Indian villagers.

**DOI:** 10.1038/bjc.1968.76

**Published:** 1968-12

**Authors:** J. J. Pindborg, F. S. Mehta, P. C. Gupta, D. K. Daftary

## Abstract

**Images:**


### PREVALENCE OF ORAL SUBMUCOUS FIBROSIS AMONG 50,915 INDIAN VILLAGERS

J. J. PINDBORG*, F. S. MEHTA, P. C. GUPTA AND D. K. DAFTARY

*From the Basic Dental Research Unit, Tata Institute of Fundamental Research, Homi Bhabha Road, Bombay 5, India*

Received for publication May 30, 1968

ORAL submucous fibrosis is a condition which has been studied in only the last 15 years, though it probably has existed for a long period of time.

With our present knowledge we may define submucous fibrosis as an insidious, chronic disease affecting any part of the oral cavity and sometimes pharynx and oesophagus. Occasionally preceded by and/or associated with vesicle formation, fibrous bands are always present, preferably in the buccal mucosa, pterygomandibular raphe and the labial mucosa. In later stages, the oral mucosa becomes stiff causing trismus and thereby inability to eat. Pigment changes—either as loss of pigment or as hyperpigmentation—are seen in most cases affecting the oral mucosa. Thus, submucous fibrosis is a clinical entity and defined as such. The condition is, however, associated with characteristic histological changes (Pindborg and Sirsat, 1966).

The condition has mainly been reported among Indians living in India, but isolated cases have been reported in Taiwan (Su, 1954), in Nepal, Thailand, South Viet-Nam, Ceylon (Pindborg and Sirsat, 1966). Among Indians living outside of India submucous fibrosis has been found in Kenya (Schwartz, 1952), Malaysia (Pindborg and Sirsat, 1966; Krishnappa, 1967), Uganda (Millard, 1966), South Africa (Shear *et al.*, 1967) and Fiji Islands (Pindborg, 1967). Isolated cases among Pakistanis and Indians living in the United Kingdom have been reported (Rowell, 1967; Kennedy and MacDonald, 1968; and Moos and Madan, 1968). Furthermore, submucous fibrosis has been diagnosed among domiciled Europeans living in Hyderabad (Rao, 1962) and in a British female living in England and married to a Pakistani (Simpson, 1968, personal communication).

It has been suggested that submucous fibrosis is a precancerous condition (Paymaster, 1956; Pindborg, 1965; Dockrat, 1967) due to its frequent association with leukoplakia and oral cancer (Pindborg *et al.*, 1967).

Epidemiological studies on the prevalence of submucous fibrosis have been done by Pindborg and co-workers and Shear *et al.* (1967). Pindborg *et al.* (1965*a*, *b*) and Zachariah *et al.* (1966) examined 35,000 urban Indians seeking the admission clinics at dental colleges in Lucknow, Bombay, Bangalore, and Trivandrum and found the following prevalence figures: 0·5%, 0·5%, 0·2% and 1·2%. Shear *et al.* (1967) who examined 1000 Indians in South Africa found a prevalence of 0·5%.

In order to compare the findings in urbanized Indians with those in rural Indians it was decided to make an epidemiological survey among villagers in

* Present address: Department of Oral Pathology, Royal Dental College, and Dental Department, University Hospital, Copenhagen, Denmark.

India. The survey also comprised a study of oral cancer and several oral precancerous conditions.

### MATERIAL AND METHODS

*Study population*

Five districts in 4 of the states in India were selected for the survey on the basis of existing prevalence of chewing and smoking habits, Fig. 1. The villages to be studied were chosen by the technique of random sampling. In the state of Bihar, 2 districts were studied because the district first chosen turned out to be inhabited by tribal groups with a specific way of life deviating from the pattern in nontribal areas. In this house-to-house survey about 10,000 individuals (all 15 years or older) were examined in each district.

*Diagnostic criteria*

*Submucous fibrosis* was diagnosed solely on clinical grounds, and only when the patients exhibited the presence of palpable fibrous bands.

*Leukoplakia* was defined as a white patch of the oral mucosa, measuring 5 mm. or more, which could not be scraped off and which could not be attributed to any other diagnosable disease. The definition does not carry any histologic connotation.

*Methods of examination*

The examinations were done by 9 Indian dentists who were trained by and calibrated to the senior author. The criteria for leukoplakia and submucous fibrosis were the same as used by the senior author in the above-mentioned surveys among urbanized Indians.

Before examination, the individuals were questioned about chewing and smoking habits. The past history with regard to oral symptoms was collected for the individuals suffering from submucous fibrosis. The examination took place in adequate natural light using two mouth mirrors. The lesions were indicated on specially designed diagrams of the oral mucosa and were photographed in colour with a Polaroid® camera. In 54 of the 63 patients with submucous fibrosis biopsies were taken; a report on the histological findings will appear later.

### OBSERVATIONS

Table I gives the prevalence figures for submucous fibrosis, leukoplakia, and oral cancer. The prevalence of submucous fibrosis varies from 0 in Singhbum in Bihar to 0·4% in Kerala. Leukoplakia varies from 0·2% in Singhbum in Bihar to 5·1% in Andhra Pradesh. The highest number of oral cancer cases was found in Kerala (10 cases) and Andhra Pradesh (7 cases). The distribution of the 63 cases according to sex and age are seen in Table II. The ratio female : male is 3 : 1. No case was found below the age of 20 years.

The oral symptoms were registered for 61 patients. From Table III it is seen that a burning sensation to spicy food was experienced in 54 patients. Next in frequency were pain, dryness of the mouth, and stomatitis. It is interesting to note that 16 patients complained of increased salivation. Twenty patients had noticed the presence of vesicles during the course of the disease.

**FIG. 1.—Map of India showing the 5 districts in the 4 states where the survey was carried out.**

TABLE I.—*Prevalence Figures for Submucous Fibrosis, Leukoplakia, and Oral Cancer Among 50,915 Indian Villagers*

| Condition | Gujarat (10,071 examined) | | Kerala (10,287 examined) | | Andhra Pradesh (10,169 examined) | | Bihar: Singhbum (10,048 examined) | | Bihar: Darbhanga (10,340 examined) | |
|---|---|---|---|---|---|---|---|---|---|---|
| | No. | Percentage | No. | Percentage | No. | Percentage | No. | Percentage | No. | Percentage |
| Submucous fibrosis | 16 | 0·2 | 36 | 0·4 | 4 | 0·04 | 0 | — | 7 | 0·07 |
| Leukoplakia | 181 | 1·8 | 266 | 2·6 | 519 | 5·1 | 23 | 0·2 | 30 | 0·3 |
| Oral cancer | 3 | 0·03 | 10 | 0·08 | 7 | 0·08 | 0 | — | 1 | 0·01 |

TABLE II.—*Distribution of the* 63 *Patients with Submucous Fibrosis According to Age and Sex*

| Age Group | Male | Female | Total |
|---|---|---|---|
| 20–29 | — | 3 | 3 |
| 30–39 | 1 | 12 | 13 |
| 40–49 | 6 | 11 | 17 |
| 50–59 | 3 | 12 | 15 |
| 60–69 | 5 | 7 | 12 |
| 70–79 | 1 | 1 | 2 |
| 80–89 | — | 1 | 1 |
| Total | 16 | 47 | 63 |

TABLE III.—*Oral Symptoms Reported for* 61 *Patients with Submucous Fibrosis*

| Symptom | Total |
|---|---|
| Burning sensation on spicy food | 54 |
| Pain | 41 |
| Dryness of the mouth | 34 |
| Stomatitis | 29 |
| Burning sensation on ordinary food | 27 |
| Ulceration | 25 |
| Burning sensation, intermittent | 23 |
| Vesicles | 20 |
| Burning sensation, continuous | 19 |
| Increased salivation | 16 |
| Referred pain | 15 |
| Numbness | 6 |

TABLE IV.—*Location of Fibrous Bands in* 63 *Patients with Submucous Fibrosis*

| Location | Number |
|---|---|
| Buccal mucosa | |
| Right | 60 |
| Left | 59 |
| Soft palate | 31 |
| Tongue | 23 |
| Labial mucosa | |
| Upper | 18 |
| Lower | 22 |
| Floor of the mouth | 18 |
| Uvula | 11 |

In Table IV the location of fibrous bands is given for the 63 cases of submucous fibrosis. The buccal mucosa is the site most frequently affected. Next in frequency are the soft palate, tongue, labial mucosa, and floor of the mouth.

Often the tongue is the seat of marked atrophy of the papillae (Fig. 2). Among the 63 cases, 24 presented a total atrophy of the tongue papillae, and 14 a partial atrophy. It means that 60% of the patients with submucous fibrosis exhibited changes in the papillary pattern of the tongue. Sixteen patients (25·4%) could not protrude the tongue beyond the muco-cutaneous junction of the lips and two beyond the incisal edges of the lower anterior teeth. Deviations from the normal oral pigmentation were observed in 23 cases. The presence of vesicles at the time of examination was noted in 6 patients.

In 8 patients (or 12·7%) the submucous fibrosis was associated with leukoplakia. Of the 10 cases with oral cancer in Kerala two also suffered from submucous fibrosis.

2

FIG. 2.—Tongue changes in a 30-year-old man, with submucous fibrosis, from Kerala. The tongue exhibits total loss of papillae and an area of retraction due to the presence of fibrous bands. The tongue cannot be stretched very much beyond the incisal edge of the lower incisor teeth. Note also the fibrotic pterygomandibular raphe in both sides and the patchy loss of pigment on the vermilion border.

### DISCUSSION

It is interesting to compare the prevalence figures for submucous fibrosis found in the present study with those reported by Pindborg and co-workers among urbanized Indians (Table V). That material was to a certain extent selected as it only comprised individuals seeking the dental colleges. They were not, however, coming because of symptoms from their submucous fibrosis, but because they wanted to get their teeth extracted or cavities filled. As in the present study, Zachariah *et al.* (1967) found the highest prevalence of submucous fibrosis in Kerala. The lowest prevalence found among urbanized Indians was in Bangalore, which is located about 1000 metres above sea level. At the present time it cannot be said whether altitude plays any role in the prevalence of submucous fibrosis.

The sex distribution in the present survey with a female : male ratio of 3 : 1 is surprising in the light of previous findings. In the largest materials published so far (Pindborg and Sirsat; 1966; Wahi *et al.*, 1966) males have dominated over females. As the present study is a house-to-house survey any selection should be excluded. Also Shear *et al.* (1967) found a predominance of females among unselected Indians in South Africa.

TABLE V.—*Prevalence Figures for Submucous Fibrosis and Leukoplakia Among 35,000 Urban Indians*

| | Bangalore (10,000 examined) | | Bombay (10,000 examined) | | Lucknow (10,000 examined) | | Trivandrum (5000 examined) | |
|---|---|---|---|---|---|---|---|---|
| | No. | Percentage | No. | Percentage | No. | Percentage | No. | Percentage |
| Submucous fibrosis | 18 | 0·2 | 50 | 0·5 | 51 | 0·5 | 61 | 1·2 |
| Leukoplakia | 166 | 1·6 | 284 | 2·8 | 328 | 3·3 | 118 | 2·4 |

The present study has not provided new information with regard to the aetiology of submucous fibrosis. The use of tobacco (Wahi *et al.*, 1966) and chillies (Pindborg and Sirsat, 1966) has been incriminated and so have vitamin deficiencies (Wahi *et al.*, 1966). It is a fact that the disease is predominantly observed among East Indians, though it has also been found in other countries of South East Asia. It is also well known that the Indians living outside Africa to a large extent keep their Indian dietary habits and that chillies are an important ingredient of the food. In the present study intolerance to spicy food was observed in 88·5% of the submucous fibrosis cases. Of the 63 cases of submucous fibrosis 31·8% did not have any chewing or smoking habit speaking against the theory that tobacco plays an important role.

It seems beyond any doubt that submucous fibrosis is most prevalent in Kerala in South India, where the oral cancer prevalence is very high. In the present study 12·7% of the submucous fibrosis cases were associated with leukoplakia, which is significantly higher than the 2·0% found in the entire survey.

This figure is lower than the 26·6% reported by Pindborg (1965) in submucous fibrosis patients from Bombay and Lucknow. The lower prevalence of leukoplakia in the present material may be explained by the fact that the 16 cases of submucous fibrosis in Gujarat were found among women, who had no chewing or smoking habits. Therefore, they lacked the agents probably responsible for inducing leukoplakia.

Of the 10 cases of oral cancer in Kerala, 3 had a simultaneous occurrence of submucous fibrosis which is in good agreement with the findings of Pindborg *et al.* (1967), *viz.*, 40% with submucous fibrosis among 100 cases of oral cancer. The results indicate a positive relationship between the two conditions. The histologic findings from the present study show a considerable number of premalignant features in the patients with submucous fibrosis thus emphasizing the precancerous nature of submucous fibrosis.

### SUMMARY

The prevalence of submucous fibrosis has been studied in 5 groups, approximately 10,000 in each, of Indian villagers in 4 states of India. The prevalence rate varied from 0 to 0·4%. Clinical data are given on the 63 cases found in the survey. A conspicuous feature is the 60% prevalence of atrophy of the tongue papillae. The etiology of submucous fibrosis is still unknown though the use of chillies seems to be associated with the development of the disease. The present findings support the hypothesis that submucous fibrosis is a precancerous condition.

The research conducted for this paper was supported in whole by funds from the National Institutes of Health, U.S. Public Health Service, under P.L. 480 research agreement No. 644,322.

The authors wish to express their profound appreciation to the dentists in the examining teams: Dr. R. B. Bhonsle, S. K. Choksi, V. V. Dandekar, Y. Mehta, V. K. Pitkar, P. N. Sihor, N. C. Shah, B. C. Shroff, P. S. Turner and S. Upadhyay.

The project is greatly indebted to Polaroid Land Corporation for an invaluable supply of cameras and films.

### REFERENCES

DOCKRAT, I. S.—(1967) *S. Afr. Cancer Bull.*, **11**, 103.
KENNEDY, T. F. AND MACDONALD, D. G.—(1968) *Br. dent. J.*, **124**, 121.
KRISHNAPPA, A.—(1967) *Dent. J. Malaysia & Singapore*, **7**, 32.
MILLARD, P. R.—(1966) *Br. J. Derm.*, **78**, 305.
MOOS, K. F. AND MADAN, D. K.—(1968) *Br. dent. J.*, **124**, 313.
PAYMASTER, J. C.—(1956) *Cancer, N.Y.*, **9**, 431.
PINDBORG, J. J.—(1965) *Bull. Wld Hlth Org.*, **32**, 748.—(1967) Report on 'Studies on Oral Leukoplakias in New Guinea and Fiji'. Submitted to the World Health Organization.
PINDBORG, J. J., CHAWLA, T. N., MISRA, R. K., NAGPAUL, R. K. AND GUPTA, V. K.—(1965*a*) *J. dent. Res.*, **44**, 615.
PINDBORG, J. J., KALAPESSI, H. K., KALE, S., SINGH, B. AND TALYERKHAN, B. N.—(1965*b*) *J. all-India dent. Ass.*, **37**, 228.
PINDBORG, J. J., POULSEN, H. E. AND ZACHARIAH, J.—(1967) *Cancer N.Y.*, **20**, 1141.
PINDBORG, J. J. AND SIRSAT, M. S.—(1966) *Oral Surg.*, **22**, 764.
RAO, A. B. N.—(1962) *Br. J. Surg.*, **50**, 23.
ROWELL, N. R.—(1967) *Br. J. Derm.*, **79**, 64.
SCHWARTZ, J.—(1952) Cited by Sirsat and Khanolkar, 1962.
SHEAR, M., LEMMER, J. AND DOCKRAT, I. S.—(1967) *S. Afr. J. med. Sci.*, **32**, 41.
SU, I. P.—(1954) *Arch. Otolar.*, **59**, 330.
WAHI, P. N., KAPUR, V. L., LUTHRA, U. K. AND SRIVASTAVA, M. C.—(1966) *Bull. Wld Hlth Org.*, **35**, 793.
ZACHARIAH, J., MATHEW, B., VARMA, N. A. R., IQBAL, A. M. AND PINDBORG, J. J.—(1966) *J. all-India dent. Ass.*, **38**, 290.